# Acute Total Hip Arthroplasty for the Treatment of Acetabular Fractures: A Retrospective Study With a Six-Year Follow-Up

**DOI:** 10.7759/cureus.10139

**Published:** 2020-08-30

**Authors:** Michail Sarantis, Sophia Stasi, Christos Milaras, Dimitrios Tzefronis, Panagiotis Lepetsos, George Macheras

**Affiliations:** 1 Orthopaedics, KAT Hospital, Athens, GRC; 2 Physiotherapy, Laboratory of Neuromuscular and Cardiovascular Study of Motion, University of West Attica, Athens, GRC

**Keywords:** acetabular fracture, reinforcement ring, jumbo acetabular cup, acute treatment, elderly, tha

## Abstract

Objectives

While open reduction and internal fixation is considered the gold standard for the treatment of acetabular fractures, it is associated with significant complications due to prolonged immobilization for elderly patients. The aim of this study was to investigate the clinical and radiological outcomes in elderly patients treated with an acute total hip arthroplasty (THA).

Patients and methods

This retrospective study included 16 patients (10 women and 6 men) with a mean age of 80.1 years suffering from a displaced acetabular fracture after a low-energy trauma. Primary THA was performed in all cases, by the same surgeon, within a three-week period after the fracture. The Burch-Schneider reinforcement ring with a cemented cup was used in 10 patients and a jumbo acetabular cup was used in 6 patients, whereas autologous bone graft was used in all cases.

Results

With a mean follow-up of 72 months, one dislocation occurred that was treated with close reduction, and one patient developed superficial site infection that was managed conservatively with antibiotics. No periprosthetic fractures, deep infections, or other adverse events were observed. One case of asymptomatic radiographic loosening was reported and treated conservatively. And autologous bone graft was well incorporated. Clinical scores were significantly improved, and all patients were able to walk independently.

Conclusions

Acute THA for the treatment of displaced acetabular fractures in elderly patients seems to be a safe option with good functional and radiological outcomes and low complication rates, offering early mobilization and weight-bearing ability to elderly patients.

## Introduction

Acetabular fractures have a reported annual incidence of around three cases per 100,000 worldwide [1,2]. Acetabular fractures are complex intra-articular injuries that occur in a bimodal distribution, typically in younger patients involved in high-energy blunt trauma, usually secondary to road traffic collisions. Contrary to young adults, in the elderly population, low-energy trauma is responsible for a high percentage of acetabular fractures due to the frequency of falls and increased prevalence of osteopenia and osteoporosis. Geriatric acetabular fractures are on the rise likely because of longer life expectancy and higher activity levels [3].

The acetabular fractures in the elderly population are often characterized by displacement of the anterior column with the separation of a quadrilateral plate fragment and comminution, and femoral head and dome impaction [4]. The optimal treatment of this subpopulation remains controversial. Nondisplaced or minimally displaced (<2 mm) fractures can be treated with non-operative methods. Conservative treatment with prolonged immobilization or even bed rest is associated with a poor outcome, and the morbidities associated therewith can be life-threatening [5]. Open reduction and internal fixation (ORIF) is considered the gold standard of treatment for displaced fractures. Restoration of congruency of the joint plays an important role in treatment outcome, although development of post-traumatic osteoarthritis can occur even after anatomical reconstruction [6]. Several authors have reported that ORIF in elderly patients is associated with poor prognosis and a higher rate of long-term complications [7]. Although ORIF can reduce the risk of developing post-traumatic arthritis, it certainly does not eliminate it, with a frequency of post-traumatic arthritis ranging from 12% to 57% [4,8]. Complex acetabular fractures and poor bone stock make ORIF technically difficult, and the result is associated with hardware failure and posttraumatic osteoarthritis [6,9,10]. THA after posttraumatic arthritis or after failed ORIF generally has inferior outcomes compared to primary THA due to osteoarthritis [11,12]. Consequently, the role of acute (early) THA has lately attracted an increased interest with clearly defined indications and promising results [13]. The appropriate initial management choice is critical due to the marked morbidity associated with revision and salvage procedures for these injuries.

The aim of this study was to investigate the clinical and radiological outcomes in elderly patients (over 75 years old) suffering from an acetabular fracture and treated with an acute (within three weeks) THA.

## Materials and methods

A retrospective study was conducted including 16 patients diagnosed with acetabulum fracture and treated with THA in our department between 2012 and 2017. The indications for THA included an impaction fracture of the femoral head with acetabular fracture, osteoporosis with impaction or comminution of the roof of the acetabulum, pre-existing osteoarthritis, or avascular necrosis. All the patients had sustained a displaced acetabular fracture (>2-mm displacement in the weight-bearing part of the joint or acetabular protrusion of the femoral head) involving the anterior column with an intact or partially intact posterior column after a low-energy trauma. Patients with cognitive dysfunction, alcohol or drug abuse, pathological fractures, inflammatory disease, or both column fractures were excluded from the study. Before the fracture occurred, all patients were able to walk independently. Fractures were classified according to Letournel [14] and Association for Osteosynthesis/Orthopaedic Trauma Association (AO/OTA) [15]. Plain radiographs and computed tomography (CT) scan with fine cuts as well as coronal/sagittal/ and three-dimensional reconstructions (including formatted images of the acetabulum with the femoral bone subtracted) was performed in all patients as a routine scan in the emergency department to identify the extent of the fracture and surrounding impaction.

All patients were operated at the same center and by the same surgeon using either a Burch-Schneider ring (Zimmer Biomet, Warsaw, IN, USA) or a jumbo acetabular cup (Zimmer Biomet). The final decision of implant use was made intraoperatively after the acetabulum exposure. The procedures were performed under spinal anesthesia. The patient was placed in the lateral decubitus position, and standard posterior approach to the hip was used [16]. After the capsulotomy, the hip was dislocated and the femoral head was resected and was used as a structural graft in case of a major peripheral acetabular defect due to severe comminution. The acetabulum was fully exposed and assessed for the fracture pattern, the extent of bone loss, and the presence or absence of pelvic discontinuity. When preparing the socket, the remaining acetabular cartilage or fibrous tissue was completely removed in order to prevent obstruction of the cup/host bone interface. The bone graft was packed into displaced fracture lines, and the quadrilateral surface bone grafting with hand-packing and reverse-reaming techniques was performed to contained defects. A Burch-Schneider reinforcement ring/antiprotrusion cage was adjusted at the center of rotation in the acetabular floor and secured to the pelvis using two to five screws in the intact part of the ileum, and the nose was inserted into the ischium. The number and position of the screws were determined by the surgeon so as to create primary stability. Then an acetabular cup for a 28-mm head was cemented into the reinforcement ring. When the jumbo acetabular cup was used, the reconstructed or grafted acetabulum was reamed carefully to produce a consistent hemispherical dome. Hemispherical reaming was performed to establish a bleeding bone bed. Eccentric or superior reaming was avoided within a deformed acetabulum and preserved the anterior/posterior acetabular walls as the cup was sequentially expanded with serial reamers. As many screws as possible to the pelvis were used to reinforce fixation of the cup. The decision for a cemented or uncemented femoral stem was based on the bone quality. Those with significant osteopenia or “stovepipe” morphology to the proximal femoral canal underwent cemented fixation.

All patients received 1 g of intravenous vancomycin 2 hours prior to surgery and 1 g post-operatively as antibiotic prophylactics. Low-molecular-weight heparin was given as thromboembolic prophylaxis for 30 days after the operation. The primary stability of the cup was good enough to allow immediate weight-bearing. On the second postoperative day, all patients were mobilized under the supervision of a physical therapist.

Patients were followed-up at six weeks, three months, six months, one year, and six years after surgery. Patients were invited to return to the clinic for a clinical evaluation and plain radiographs. At the follow-ups, information on any adverse events was collected, clinical and radiological investigations were performed, and functional and health-related quality-of-life questionnaires were completed. All patients were asked to complete an Oxford Hip Score (OHS) questionnaire [17], a reliable, validated 12-item questionnaire that assesses functional ability and pain from the patient’s perspective. Hip function at the follow-ups was evaluated using the Harris Hip Score (HHS) comprising four dimensions: pain, function, absence of deformity, and range of motion. A score of 70 was regarded as poor, 70-79 as fair, 80-89 as good, and 90-100 as excellent. The health-related quality of life was assessed before the fracture occurred and at the follow-ups using a generic instrument, the health part of EuroQol (EQ-5D index score). The score ranges from 0, indicating the worst possible health state, to 1, which indicates the best possible health state.

The presence of heterotopic ossification (HO) was assessed using all three Judet views and classified according to Brooker et al. [18]. Osteolysis of the acetabulum was evaluated according to the zones described by DeLee and Charnley [19]. Loosening of the femoral component was assessed using the criteria of Harris and McGann [20], and the location of any osteolytic lesions was evaluated using the zones described by Gruen et al. [21]. For this study, we defined acetabular loosening as circumferential radiolucent lines > 2 mm and/or evidence of migration.

## Results

Sixteen patients were initially included; two patients died at 9 and 18 months, respectively, after the operation due to unrelated causes, leaving 14 patients (eight females and six males) for the final follow-up investigation. Characteristics of the study population are shown in Table 1. The mean age was 80.1 years (range: 76-89 years), and the mean bone mass index was 27.8. The mean follow-up was 71.8 months. The mean time elapsed between patient trauma and operation was 13 days (range: 3-21 days). Examination of type of trauma revealed two cases of non-vehicle traffic accident (NVTA), five cases of vehicle traffic accident (VTA), five cases of a fall from a height (FDFH), and four cases of same level fall (SLF). Fractures were classified according to Letournel and AO/OTA as anterior column (62-A3) (n = 9), anterior column and posterior hemitransverse (62-B3) (n = 5), or transverse juxtatectal (62-B1) (n = 2). The mean operating time was 122 minutes (range: 101-162 minutes), and the mean intraoperative blood loss was 231 mL (range: 125-337 mL).

**Table 1:** Characteristics of the study population BMI, bone mass index; NVTA, non-vehicle traffic accident; VTA, vehicle traffic accident; FDFH, fall down from height; SLF, same level fall; AO/OTA, Association for Osteosynthesis/Orthopaedic Trauma Association

Cases (n)	16
Female (%)	62.5%
Mean age (years)	80.1 (range 76–89)
Mean BMI (kg/m^2^)	27.8 (range 23–33)
Mean follow-up (months)	71.8 (range 60–78)
Mean surgical time (minutes)	122 (range 101–162)
Mean hospitalization (days)	9.1 ± 1.1 (range 7–15)
Mortality rate	12.5% at six years
Reason for acetabular fracture	NVTA (12.5%), VTA (31.25%), FDFH (31.25%), SLF (25 %)
AO/OTA Classification	62-A3 (n = 9), 62-B3 (n = 5), 62-B1 (n = 2)
Surgical approaches	Posterior approach: 16 patients

Diagnosed osteoporosis under treatment with bisphosphonates was present only in one female patient, and osteopenia without treatment was present in four female patients. From patient medical history, the mean dual-energy X-ray absorbtiometry (DXA) scan T-score before fracture in the five female patients was -2.1 (range: -1.5 to -2.8). Osteoarthritis was present during the routinely assessed pelvis radiographs in 13 out of 16 patients, with a mean Kellgren-Lawrence score of 2.2 (range: 1-3).

**Figure 1:**
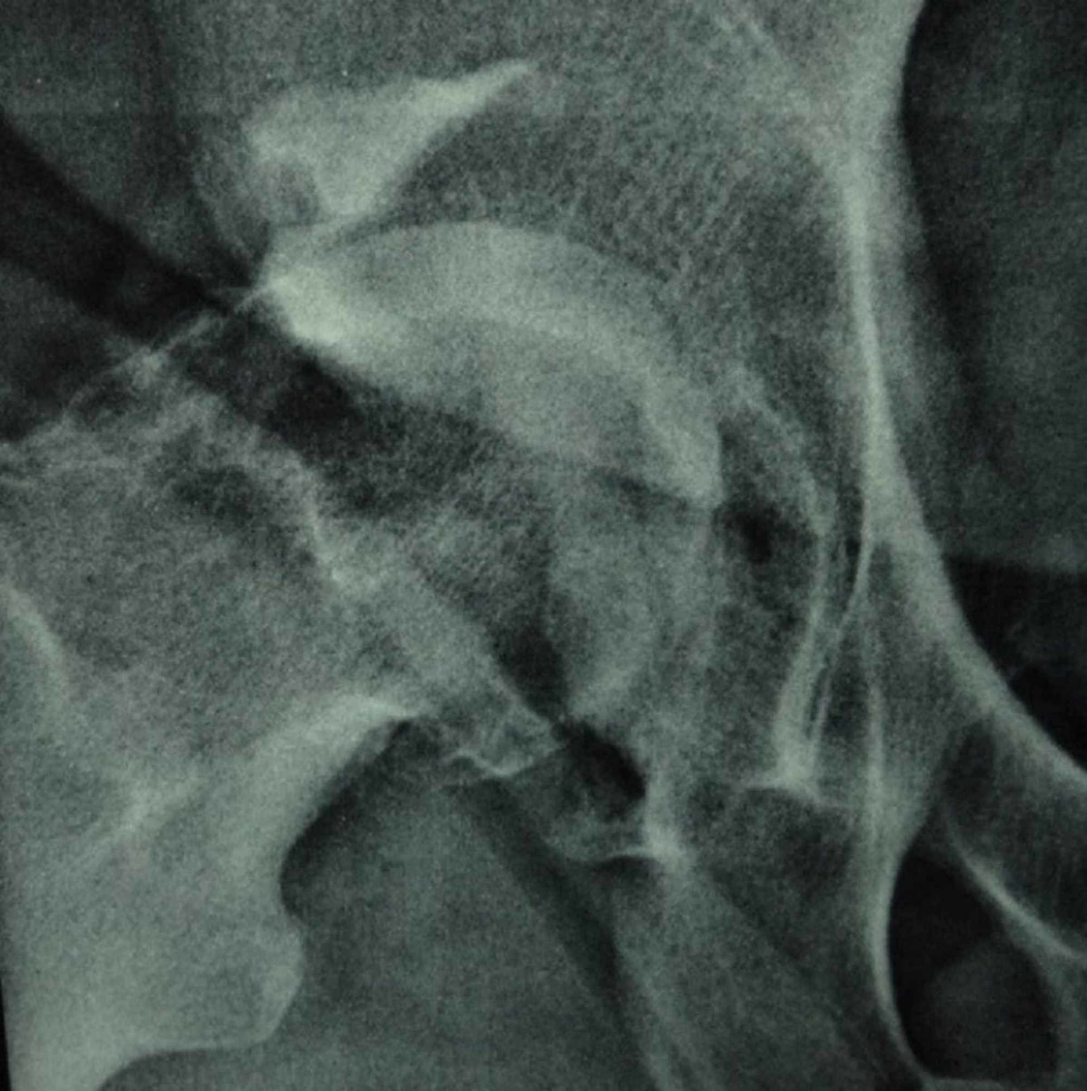
Preoperative X-ray of an 81-year-old patient with an acetabular fracture

**Figure 2:**
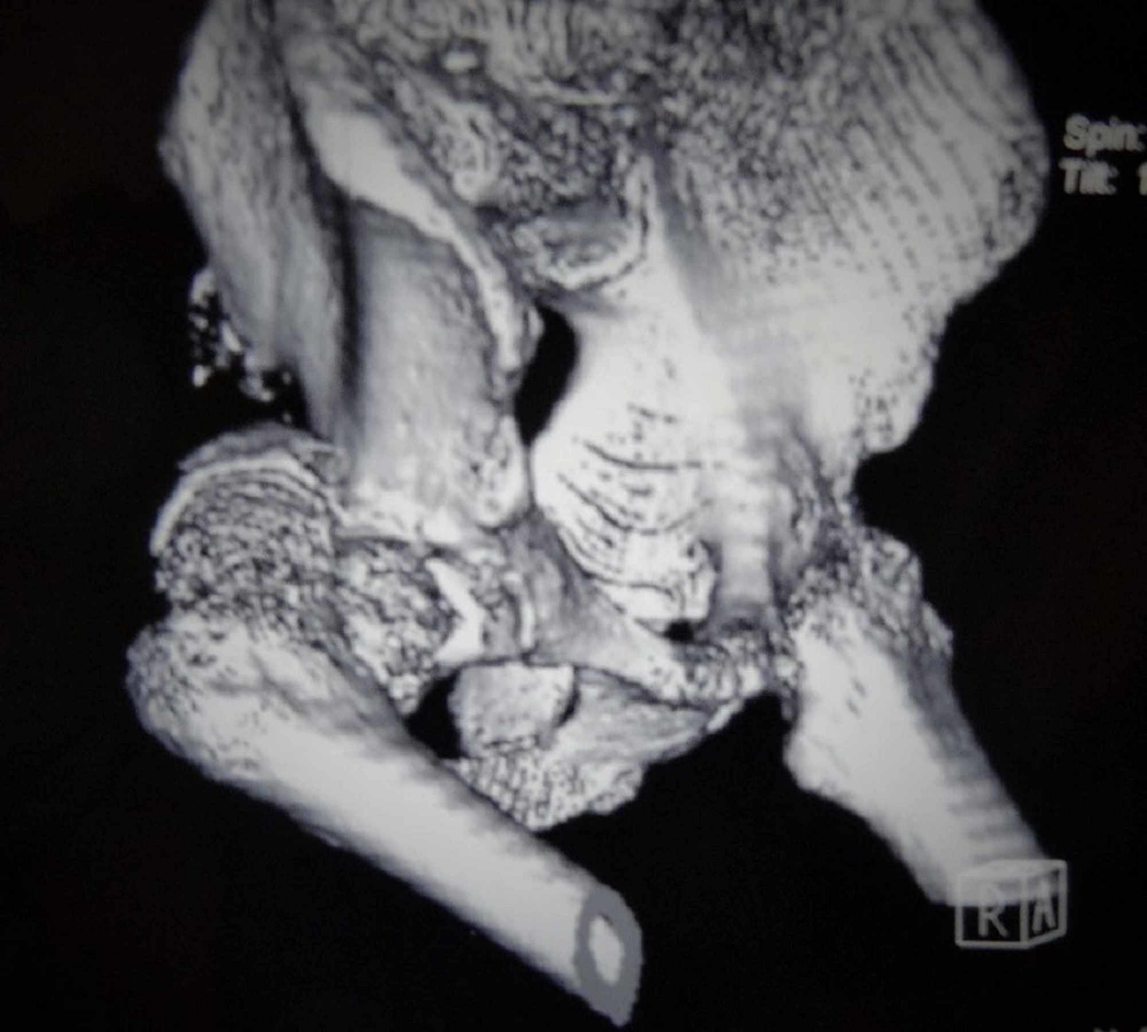
Preoperative CT reconstruction of the same patient

**Figure 3:**
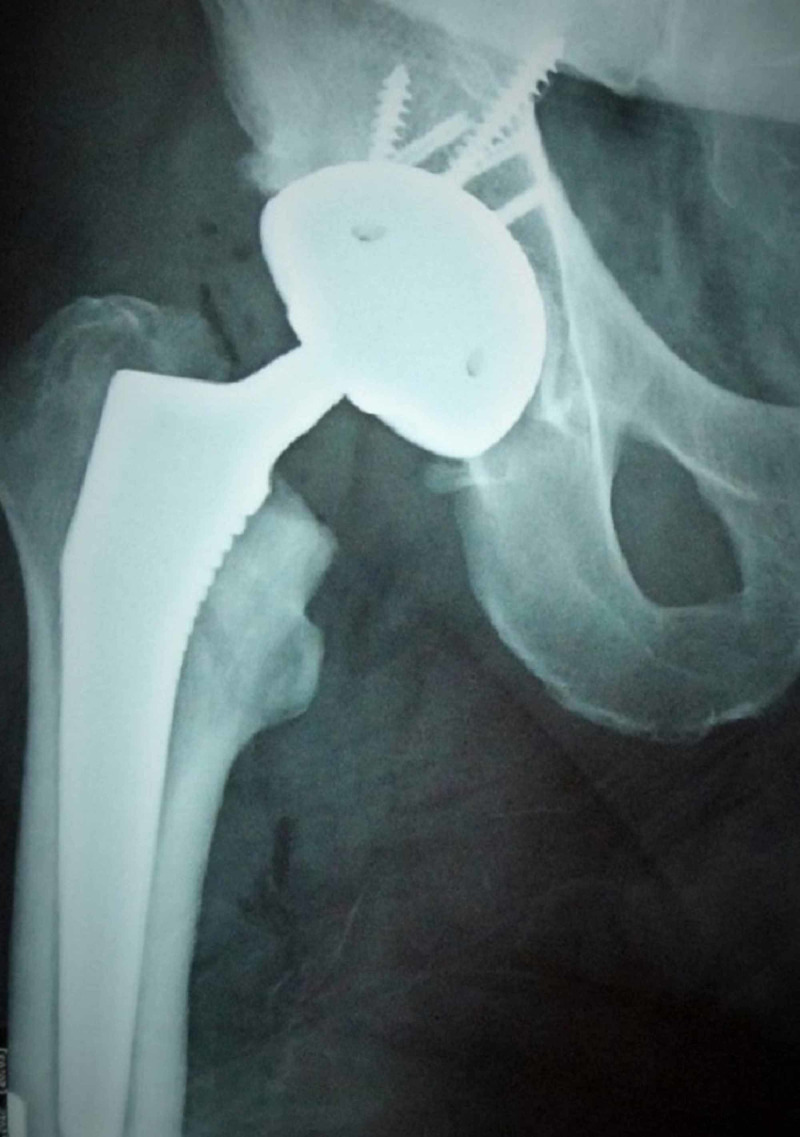
Radiograph of the same patient three months postoperatively

**Figure 4:**
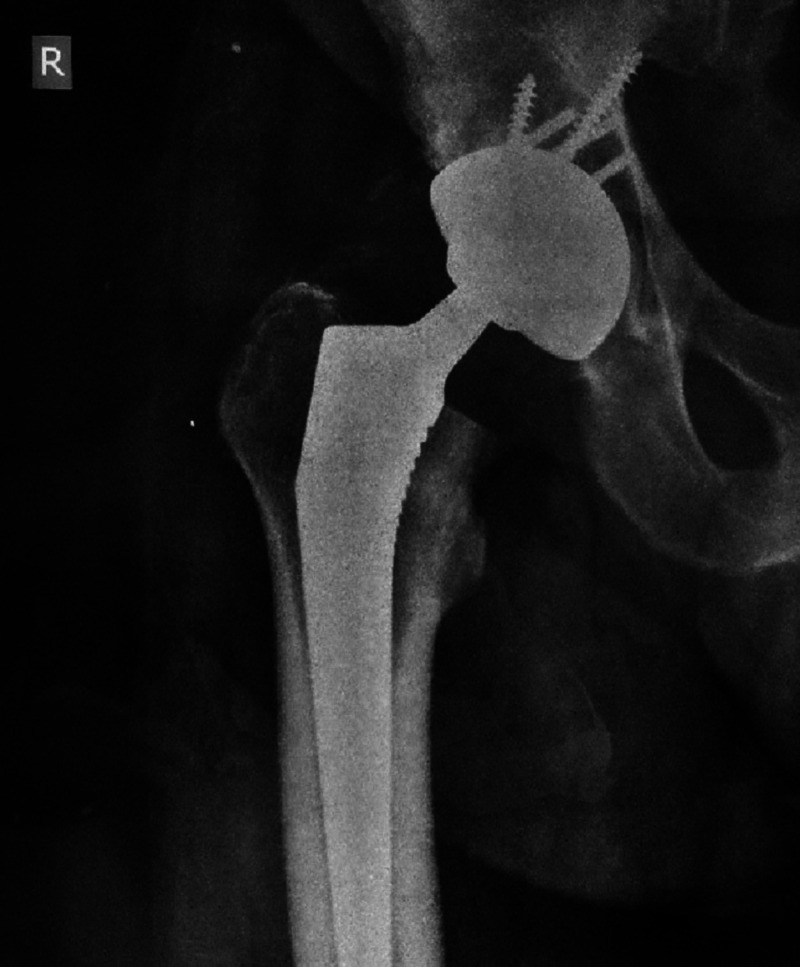
Radiograph of the same patient one year postoperatively

**Figure 5:**
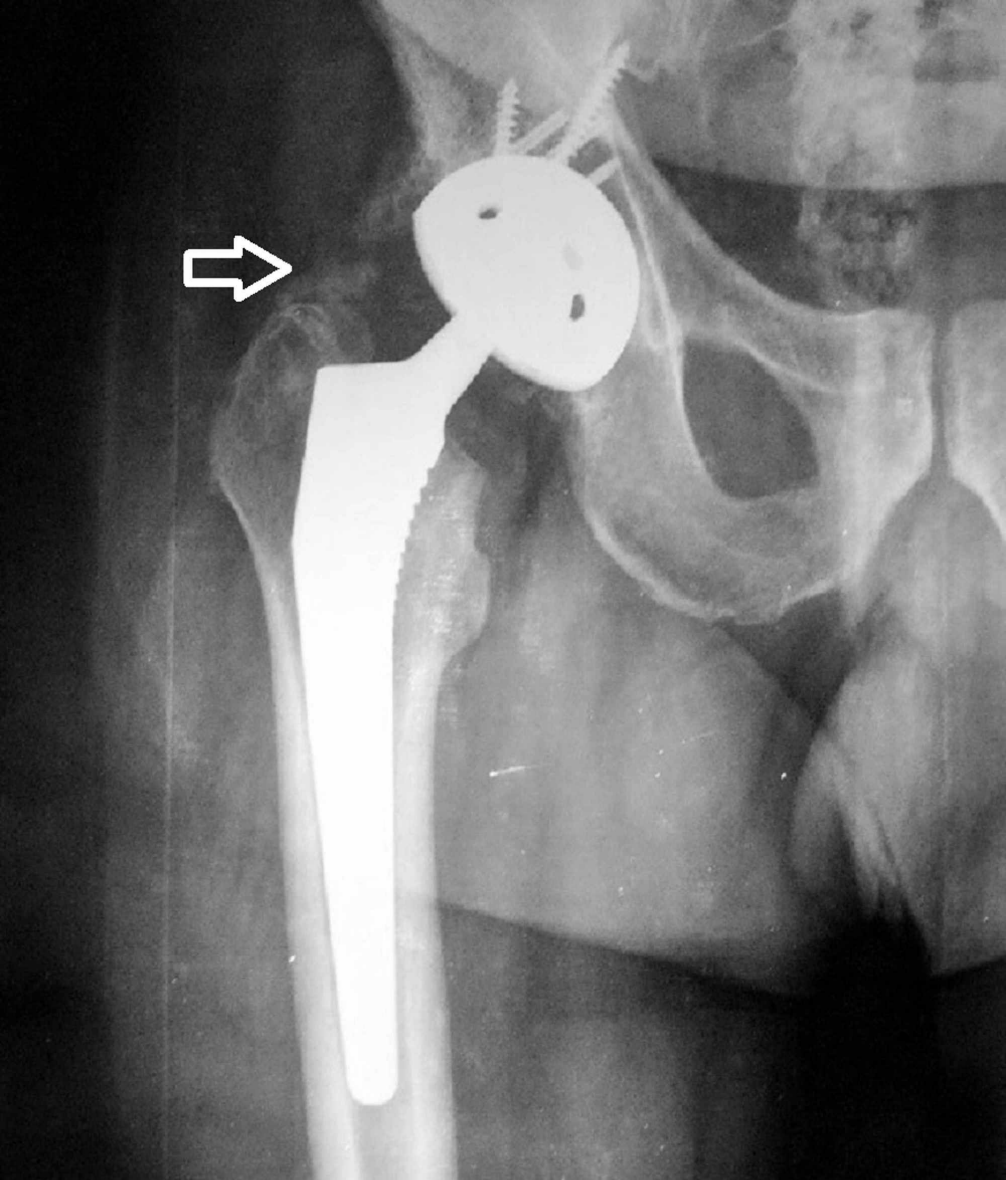
Radiograph of the same patient six years postoperatively (heterotopic ossification, Brooker III type, as marked by the white arrow)

**Figure 6:**
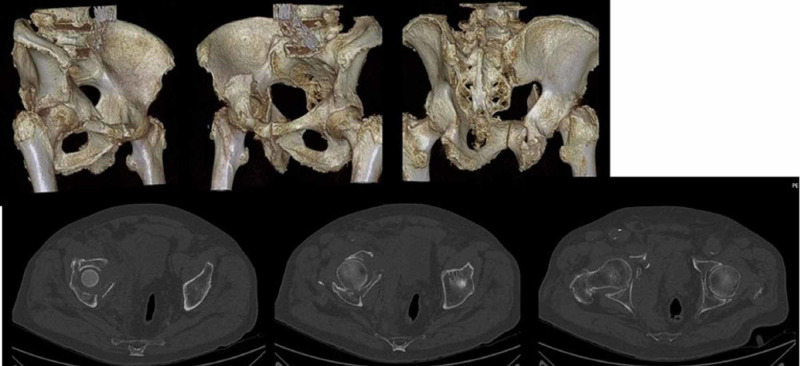
Preoperative CT and reconstruction of an 83-year-old patient with an acetabular fracture

**Figure 7:**
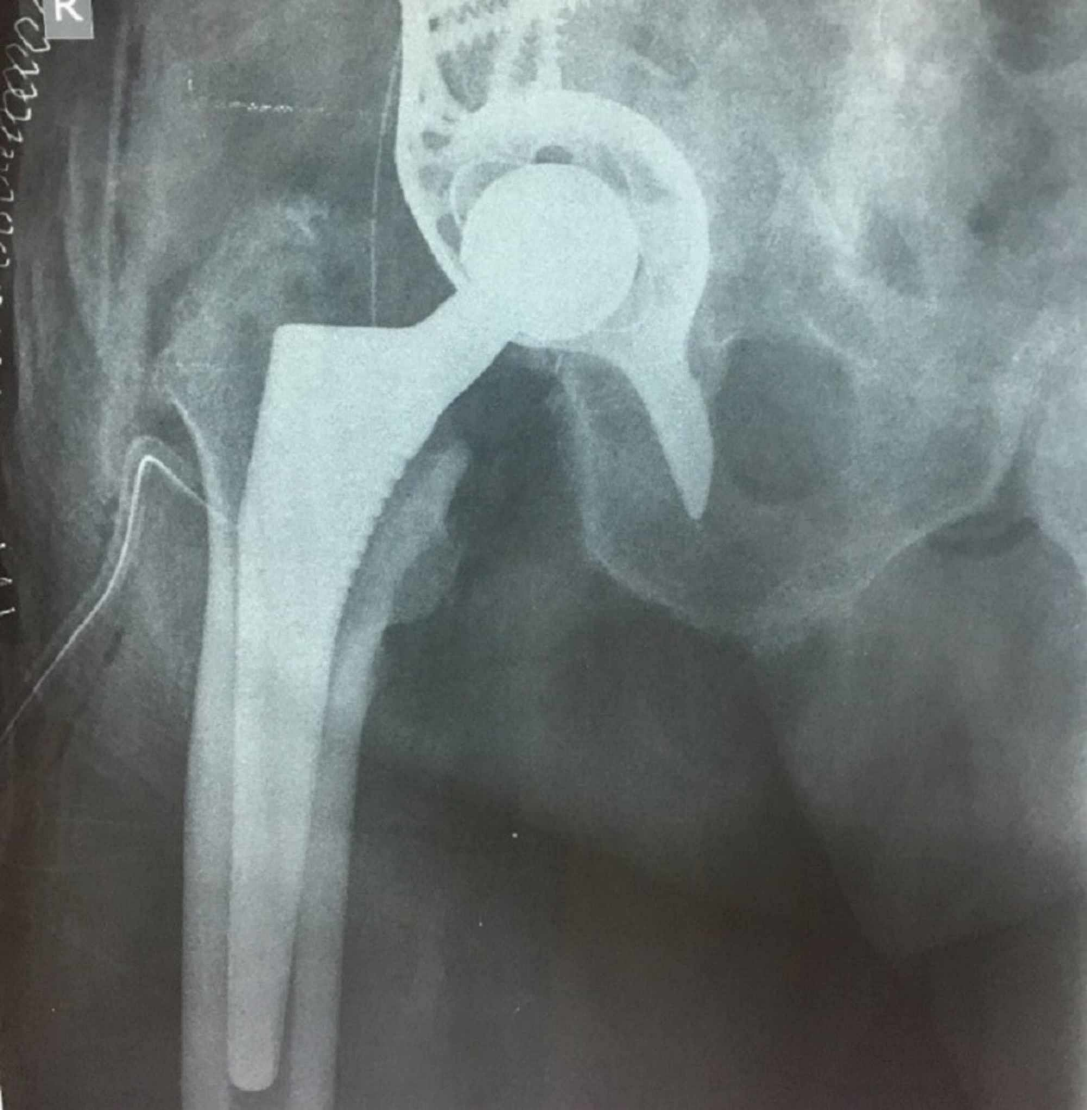
Radiograph of the same patient postoperatively

**Figure 8:**
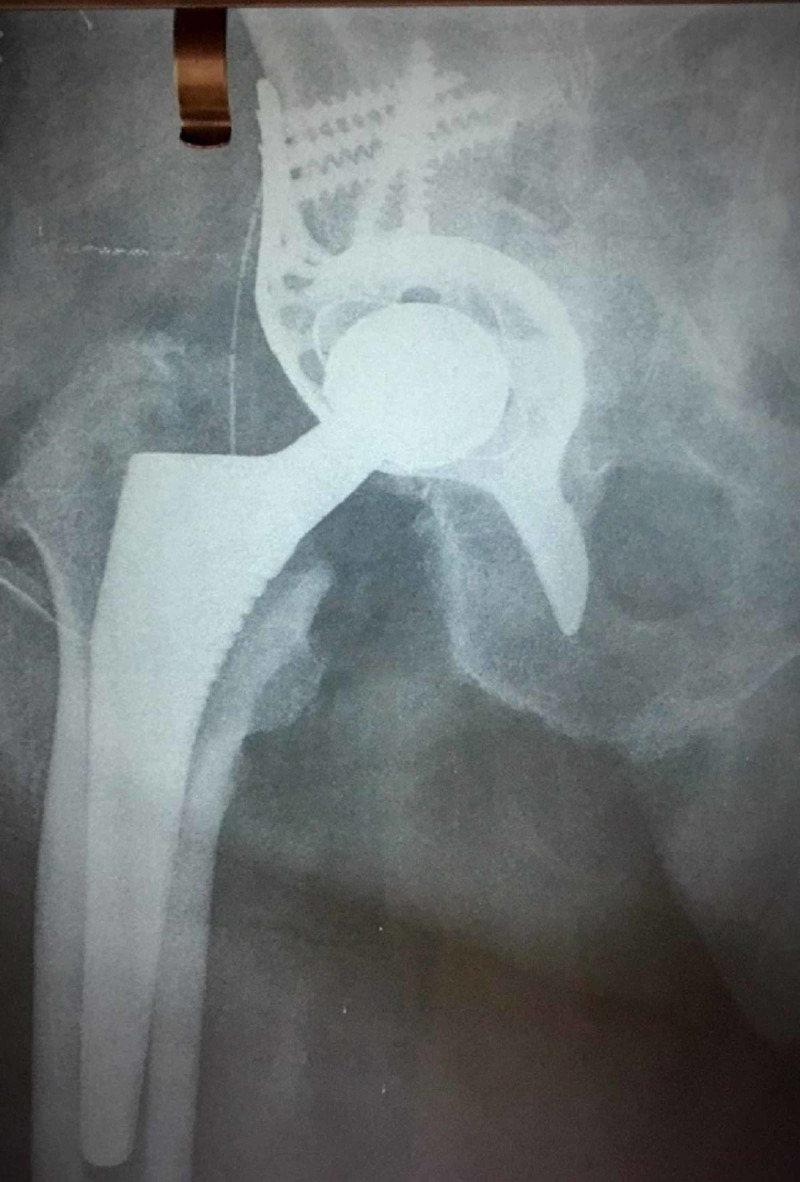
Radiograph of the same patient one year postoperatively

A jumbo acetabular cup (Figures 1-5) was used in 6 out of 16 patients, and a Burch-Schneider reinforcement ring/antiprotrusion cage (Figures 6-8) was used in the rest 10 patients. Out of 16 patients, 12 (75%) were treated with an uncemented proximally porous-coated femoral stem using standard canal preparation techniques. Four (25%) patients were treated with a cemented femoral stem using a third-generation cementation technique.

Twelve patients (10 patients were treated with a reinforcement Burch-Schneider ring and 2 patients with the jumbo acetabular cup) were instructed to partial weight-bearing with crutches or a walker for three weeks and advanced to full-weight bearing as tolerated under the supervision of a physical therapist at the sixth week after surgery. Four patients (all were treated with the jumbo acetabular cup) were restricted to touch-down weight-bearing for three weeks, partial weight-bearing for another three weeks, and weight-bearing as tolerated after the sixth postoperative week.

Two patients aged 79 and 81 years died 9 and 18 months, respectively, after surgery due to unrelated reasons. The overall mortality rate of our patients during the 72-month period was 12.5%. One patient suffered from deep vein thrombosis without pulmonary embolism and was treated successfully with apixaban orally. One patient suffered from superficial infection of the surgical site and was treated conservatively with oral antibiotics. One patient previously treated with a Burch-Schneider cage had dislocation two months after the operation and was treated by closed reduction and hip abduction brace for six weeks. There were no periprosthetic fractures, deep site infections, or nerve palsies detected.

**Figure 9:**
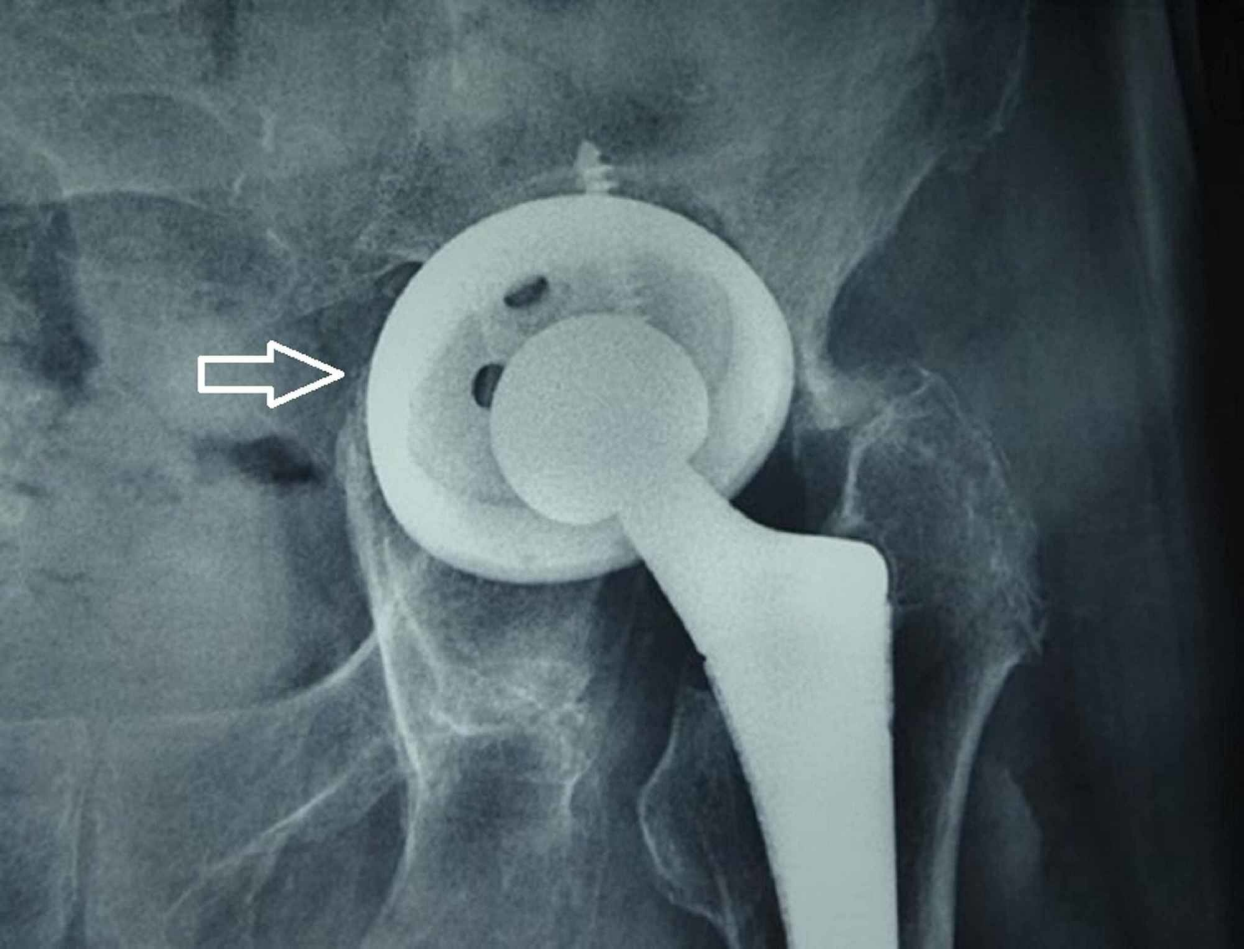
Asymptomatic aseptic loosening of the acetabular component in an 87-year-old patient at the six-year follow-up, as shown by the white arrow

There was no subsidence or loosening of the femoral component in the serial radiographs. As far as the acetabular component was concerned, one case of radiological aseptic loosening of the jumbo acetabular cup was detected. The patient, an 87-year-old male at the time of the final follow-up, was asymptomatic, walking with just one stick as walking aid, and unwilling for further surgical management (Figure 9). There was no cup migration or loosening of the Burch-Schneider cage cases. Two patients developed HO during the first months after the operation. At the one-year follow-up, one patient displayed a Brooker class I HO and one patient displayed a Brooker class II HO on the radiographs of the injured hip. At 72 months, the HO remained unchanged, class I in one patient, and progressed from class II to class III in one patient. None of our patients were treated with an initial prophylactic anti-inflammatory drug for HO prevention.

At the last follow-up, all the patients were able to walk independently with or without walking aids. Of the 14 patients, 13 used no walking aid for indoor and outdoor walking, whereas 1 used just one stick. In the 14 patients who completed the OHS (Table 1), the average score was 17 (range: 12-32). Seven (50%) patients had an excellent score, five (35%) patients had a good score, and two had a fair outcome, with a total of 12 (85%) patients reporting a good to excellent outcome. The mean EQ-5D index score was impaired from 0.89 preoperatively to 0.65 at the first follow-up (p = 0.02). It increased slightly over time at one year (mean: 0.68), but at the four-year follow-up, it was still lower (mean: 0.65) compared with the preoperative score. The mean HHS improved from fair (mean: 75.6) at three months, to good (mean: 87.5) at six months, to excellent (mean: 91.8) at one year, and was finally considered to be good (mean: 88.3) at four years. The mean total ranges of motion (flexion, internal rotation, external rotation, abduction, and adduction) at the follow-ups were 181 (range: 130-225) at three months, 202 (range: 160-245) at six months, 220 (range: 145-255) at one year, and 222 (range: 155-300) at four years.

## Discussion

Acetabular fixation in the face of significant osteopenia, prior irradiation to the pelvis, and other situations with poor bone quality have traditionally posed a challenge to the arthroplasty surgeon. Non-operative treatment and prolonged immobility have been related with serious complications such as decreased muscles strength, deep venous thrombosis, pulmonary embolism, postural hypotension, decreased cardiac function, urinary retention, constipation, pressure ulcers, impaired pulmonary function, bone reabsorption, osteoporosis aggravation, and psychological dysfunction with depression [22]. The average number of days in the hospital following non-operative acetabular fracture is high, ranging from 9.3 to 45 days. Patients who were not self-sufficient prior to fracture had a longer hospital stay [23].

Matta stated that key factors for the results after ORIF of acetabular fractures are absolute anatomical reduction, fracture complexity, and patient age [6]. Kreder et al. proposed acute THA for patients more than 50 years old with marginal impaction and wall comminution due to the high rate of failure after ORIF as initial treatment [10]. Furthermore, Carroll et al. reported that 31% of patients aged more than 55 years old with acetabular fracture treated with ORIF needed reoperation with THA [22]. In the presence of these results, this study aimed to evaluate the results of immediate THA in elderly patients with acetabular fracture.

Obviously, alternative methods to ORIF alone have to be considered for many elderly patients. A combination of ORIF and acute THA at the same time was performed by Lin et al. [24] on 33 patients (mean age: 66 years) with restricted weight-bearing for six weeks. At the last follow-up (mean follow-up period of 5.6 years), 93% of patients reported good to excellent functional results without loosening. THA combined or not with internal fixation has been proposed as a reliable one-operation option allowing early mobilization [25]. Stable fixation of the acetabular cup is crucial when the treatment choice is THA. Mouhsine et al. reported a study including 18 patients treated with cable fixation and an acute THA, and the cup migration on three months post-operative was 2.3 mm superiorly and 2 mm medially [25,26]. Mears and Velyvis studied 57 patients treated with an acute THA, and the acetabular cup was stabilized with structural autograft, screws, or cables [13].

Another study by De Bellis et al. reported indications for early THA on acetabulum fracture of complex fracture according to Letournel and Judet, osteoarthritis in hips, femur head fracture, pathological fracture, bad bone quality, or fractures that cannot be reconstructed [27]. Mears and Velyvis added these criteria to the indications: severe impaction, wide femur head abrasion, acetabular impaction more than 30% of its surface, multipartite acetabular fracture, and more [13]. Relative indications are reported as delayed case, medical comorbidities, obesity, and senility. In the present study, 5 patients had a simple fracture and 12 patients had a complex fracture. One of these simple fracture cases had severe osteoarthritis and two had osteoarthritic changes; however, these patients were elderly [7,26].

Although the use of reinforcement rings is well documented for hip arthroplasty revision surgery [28,29], there are only a limited number of studies that discuss its use for acetabular fractures. Tidermark et al. presented good results in 10 elderly patients with acetabular fracture treated acutely with a reinforcement ring without any sign of loosening after 38 months of follow-up [7]. Enocson and Bloomfeldt [29] presented remarkable results in a prospective study of 15 elderly patients (mean age of 76 years) with acetabular fracture treated acutely with a reinforcement ring without any sign of loosening after four-year follow-up. Mears and Velyvis assessed 57 acute THAs in the setting of displaced acetabular fractures and found that after 2 to 12 years of follow-up, there were no cases of loosening or osteolysis, and only one case was revised for recurrent dislocations. The authors concluded that THA after acute acetabular trauma can restore function and relieve pain [13].

In our series, we recorded good clinical, radiological, and patient-related outcomes, without the presence of serious adverse events after surgical treatment of acetabular fracture using a primary reinforcement ring, autologous bone graft, a cemented THA, or a jumbo acetabular cup. Acute surgical treatment of these fractures, when possible, allows early mobilization, minimizing the risk of serious adverse events associated with prolonged immobilization. 

The main advantage of our study is the thorough and precise evaluation of a specific age group in a relatively long follow-up time with both clinical and radiological measurements. Weakness of our study is its retrospective design and the small study population. Furthermore, reinforcement ring is not always suitable for all kinds of patients such as in those with complex acetabular fractures.

## Conclusions

Acetabular fractures in the elderly have significant complications. Acute management of these fractures and early mobilization of elderly patients play an important role in patient rehabilitation and quality of life. The main finding of our study was that the treatment of the displaced acetabular fractures in elderly patients using a primary reinforcement ring or a jumbo acetabular cup, autologous bone graft, and a THA seems to be a safe option with good functional and radiological outcomes. Further investigation with randomized studies and larger population or comparison groups are needed to clarify the role of THA in the treatment of acetabular fractures, especially in the elderly.
